# Association Analysis of Genetic Variants in the Myosin IXB Gene in Acute Pancreatitis

**DOI:** 10.1371/journal.pone.0085870

**Published:** 2013-12-30

**Authors:** Rian M. Nijmeijer, Hjalmar C. van Santvoort, Alexandra Zhernakova, Steffen Teller, Jonas A. Scheiber, Carolien G. de Kovel, Marc G. H. Besselink, Jeroen T. J. Visser, Femke Lutgendorff, Thomas L. Bollen, Marja A. Boermeester, Ger T. Rijkers, Frank U. Weiss, Julia Mayerle, Markus M. Lerch, Hein G. Gooszen, Louis M. A. Akkermans, Cisca Wijmenga, on behalf of the Dutch Pancreatitis Study Group

**Affiliations:** 1 Department of Surgery, University Medical Center Utrecht, Utrecht, the Netherlands; 2 Complex Genetics Group, Department of Medical Genetics, University Medical Center Utrecht, Utrecht, the Netherlands; 3 Department of Genetics, University of Groningen, University Medical Center Groningen, Groningen, the Netherlands; 4 Department of Internal Medicine A, Ernst-Moritz-Arndt University, Greifswald, Germany; 5 Department of Surgery, Academic Medical Center, Amsterdam, the Netherlands; 6 Department of Cell Biology, Immunology Section, University of Groningen, University Medical Center Groningen, Groningen, the Netherlands; 7 Department of Radiology, St Antonius Hospital, Nieuwegein, the Netherlands; 8 Department of Operating Room/Evidence Based Surgery, Radboud University Nijmegen Medical Center, Nijmegen, the Netherlands; 9 Department of Medical Microbiology and Immunology, St Antonius Hospital, Nieuwegein, the Netherlands; Odense University hospital, Denmark

## Abstract

**Introduction:**

Impairment of the mucosal barrier plays an important role in the pathophysiology of acute pancreatitis. The myosin IXB (MYO9B) gene and the two tight-junction adaptor genes, PARD3 and MAGI2, have been linked to gastrointestinal permeability. Common variants of these genes are associated with celiac disease and inflammatory bowel disease, two other conditions in which intestinal permeability plays a role. We investigated genetic variation in MYO9B, PARD3 and MAGI2 for association with acute pancreatitis.

**Methods:**

Five single nucleotide polymorphisms (SNPs) in *MYO9B*, two SNPs in *PARD3*, and three SNPs in *MAGI2* were studied in a Dutch cohort of 387 patients with acute pancreatitis and over 800 controls, and in a German cohort of 235 patients and 250 controls.

**Results:**

Association to *MYO9B* and *PARD3* was observed in the Dutch cohort, but only one SNP in *MYO9B* and one in *MAGI2* showed association in the German cohort (p < 0.05). Joint analysis of the combined cohorts showed that, after correcting for multiple testing, only two SNPs in *MYO9B* remained associated (rs7259292, p = 0.0031, odds ratio (OR) 1.94, 95% confidence interval (95% CI) 1.35-2.78; rs1545620, p = 0.0006, OR 1.33, 95% CI 1.16-1.53). SNP rs1545620 is a non-synonymous SNP previously suspected to impact on ulcerative colitis. None of the SNPs showed association to disease severity or etiology.

**Conclusion:**

Variants in *MYO9B* may be involved in acute pancreatitis, but we found no evidence for involvement of *PARD3* or *MAGI2*.

## Introduction

Acute pancreatitis is an acute inflammatory condition of the pancreas, resulting in over 200,000 hospital admissions in the United States each year [1]. In most patients, it is caused by gallstone disease or alcohol abuse [2], while genetic factors are thought to contribute to disease susceptibility and may influence the clinical course of the disease [3,4]. In 20% of patients, acute pancreatitis runs a severe clinical course associated with high morbidity rates and mortality of up to 30% [5]. Nearly all the deaths are associated with infectious complications, such as bacteremia and infection of pancreatic necrosis [6,7]. To date, few studies have revealed any significant association between genetic factors and acute pancreatitis, but these studies involved relatively small cohorts. They investigated over 30 candidate genes, of which only one (*SPINK1*) showed consistent association with acute and recurrent acute pancreatitis [8-12].

Failure of the gastrointestinal mucosal barrier plays an essential role in the course of acute pancreatitis, as it allows for bacterial translocation, which in turn may lead to infectious complications [13-16]. Although little is known about the exact pathophysiology of mucosal barrier failure in acute pancreatitis, it may also contribute to the development of the initial disease. Tight junction failure within the pancreas has been shown to be an extremely early event in the development of experimental acute pancreatitis in mice [17] and rats [18,19]. In a caerulein model of acute pancreatitis in rats, disruption of the actin cytoskeleton and tight junctions resulted in increased paracellular permeability [18,20]. 

Genetic associations have recently been reported for two other inflammatory conditions in which intestinal permeability plays a pathophysiological role; these are celiac disease (CD) and inflammatory bowel disease (IBD). CD and its complications have been associated to both myosin IXB (*MYO9B*) and to two tight-junction adaptor genes *PARD3* and *MAGI2* [21-24], whereas IBD has repeatedly been associated to *MYO9B* [25-29] and once to *MAGI2* [23]. All three proteins are hypothesized to play a role in tight junction assembly and positioning of the tight junctions in the membrane regions of the cell, and could thus possibly play a role in intestinal barrier function [25,30-33]. In addition to CD and IBD, *MYO9B* has also been associated with susceptibility to type 1 diabetes mellitus in a Spanish cohort [34]. We know the intestinal barrier is impaired in type 1 diabetes [35-37]. Moreover, the BioBreeding diabetes prone (BBDP) rat model of diabetes, in which spontaneous development of autoimmune type 1 diabetes occurs and which is used to study the mechanisms of diabetes pathogenesis, showed an increase in intestinal permeability, even before the onset of clinical diabetes [38,39]. 

Based on these genetic association studies in diseases with a compromised intestinal barrier, we hypothesized that polymorphisms in these three genes involved in mucosal barrier function might also be associated with acute pancreatitis. We therefore adopted a candidate gene approach to test genetic variants in *MYO9B*, *PAR3D* and *MAG12* for their potential association with acute pancreatitis in two independent cohorts: a Dutch cohort of 387 patients and more than 800 controls, and a German cohort of 235 patients and 250 controls. 

## Methods

### Cohorts

The Dutch cohort consisted of 387 patients with acute pancreatitis and over 800 random blood bank controls. This genetic association study was part of a multicenter, randomized controlled trial (trial registry number ISRCTN38327949) [40], during which patients with a first episode of acute pancreatitis were included in a prospective database. The cohort comprised 188 randomized patients and 199 patients with acute pancreatitis who had been screened for eligibility for the PROPATRIA trial, but who were not randomized [40]. Acute pancreatitis was defined as abdominal pain in combination with a greater than three-fold elevation of serum amylase or lipase concentrations [40]. Severe acute pancreatitis was defined as acute pancreatitis with organ failure and/or local complications [40]. Infectious complications were defined as infected pancreatic necrosis, bacteremia, pneumonia, urosepsis, or infected ascites [40]. All patients or their legal representatives gave written informed consent and the ethics review boards of all 15 participating hospitals approved the protocol for this part of the study (Ethics S1). Clinical data on the severity of disease and outcome for all patients were available from the PROPATRIA database (Table 1) [40]. 

**Table 1:** Clinical characteristics of the two cohorts of patients with acute pancreatitis.

**Characteristic**	**Dutch acute pancreatitis patients (n = 387)**	**German acute pancreatitis patients (n = 235)**
Male	207 (53.3%)	122 (51.9%)
Age (years, mean ± 1 SD)	56.7 (± 17.6)	52.5 (± 19.5)
*Etiology of pancreatitis*		
Biliary	209 (54%)	94 (40%)
Alcohol	72 (19%)	65 (28%)
Medication	14 (4%)	3 (1%)
Hypertriglyceridemia	3 (1%)	1 (0.5%)
Other	17 (4%)	52 (22%)
Unknown	72 (18%)	20 (8.5%)
*Severity of pancreatitis (median, IQR)*		
APACHE-II score^*^	7.0 (4.0-10.0)	5.0 (2.0-7.0)
Imrie score	2.0 (1.0-4.0)	1.0 (0-1.0)
CRP, highest value in first 48 hrs (mg/L)	192 (81-295)	88 (24-164)
Severe acute pancreatitis^‡^	104 (27%)	15 (6%)
Necrotizing pancreatitis^#^	84 (22%)	9 (4%)
*Complications*		
Infections	93 (24%)	13 (6%)
Positive blood culture	56 (15%)	10 (4%)
Organ failure during admission	58 (15%)	8 (3%)
Multi-organ failure during admission	30 (8%)	0
Mortality	20 (5%)	0

^*^ Highest score on day of admission

^‡^ Organ failure and/or necrosis

^#^ Defined as: pancreatic parenchymal necrosis demonstrated on contrast-enhanced computed tomography scan

CRP, C-reactive protein; IQR, interquartile range; SD, standard deviation

Genotype data from two control cohorts were used [21,23,25]. For the single nucleotide polymorphism (SNP) typing of *MYO9B*, the controls were random hospital controls (n = 220) [21] and Dutch blood bank donors from Utrecht, Leiden and Amsterdam (n = 1323) [21,25]. For the two tight junction adaptor genes (*PARD3* and *MAGI2*), only a subset of the controls was used (n = 848) [23]. Characteristics of the control groups have been described previously [21,23,25]. All control genotypes were in Hardy-Weinberg equilibrium (data not shown, p > 0.05).

The second cohort comprised 235 German patients with acute pancreatitis and 250 German controls. The patients were prospectively enrolled in the ProZyt study [41,42]. The definitions used for acute pancreatitis and for severe acute pancreatitis were the same as for the Dutch cohort. Clinical data on the severity of disease and outcome for all patients were available from the ProZyt Study database [41,42]. All patients gave their written informed consent and the ethics review board of Greifswald University, Greifswald, Germany, approved the protocol for the study. The German controls were healthy blood bank donors (n = 250). All control genotypes were in Hardy-Weinberg equilibrium (data not shown, p > 0.05).

For the current genetic association study, we took peripheral blood samples from each patient. These were centrifuged at 3,000 rpm for 10 minutes and the plasma and cell pellets were separated and stored at -80°C. Genomic DNA was extracted from the cell pellets using DNA isolation kit I from the Magna Pure LC (initial cohort, Roche Diagnostics, Indianapolis, USA) or the Quick-gDNA MiniPrep Kit (follow-up study, Zymo Research, Irvine, California, USA).

### SNP selection and genotyping

We selected five tag SNPs in *MYO9B* that had shown association with CD or IBD [21,25] (rs2305767, rs1457092, rs2305764, rs7259292 and rs1545620; Applied Biosystems, Foster City, California, USA). We also selected five SNPs from the two tight junction adaptor genes (three in *MAGI2* and two in *PARD3*) that were associated with CD and ulcerative colitis (rs10763976, rs4379776, rs6962966, rs9640699, and rs1496770) [23]. 

Genotyping of the two cohorts was performed independently. The Dutch cohort was genotyped in the Complex Genetics Group Laboratory, University Medical Center Utrecht, the Netherlands. The German cohort was genotyped in the Laboratory for Molecular Gastroenterology, Department of Medicine A, Greifswald Hospital, Germany. Genotyping was done using TaqMan assays (Applied Biosystems) and the genotypes were analyzed using a TaqMan 7900 HT (Applied Biosystems). Haplotypes were constructed using Haploview v4.2 [43]. 

### Statistical analysis

For continuous values of patient characteristics (Table 1), normally distributed data were presented as mean and standard deviations (SD); all non-normally distributed data were presented as medians with an interquartile range (IQR). The association study (Table 2) was analyzed using the two-tailed chi squared test for independence of case vs. control alleles in PLINK v1.07 (http://pngu.mgh.harvard.edu/purcell/plink/) [44]. For the joint analysis, allele counts for the Dutch and German cohorts were combined and a Cochran-Mantel-Haenszel analysis was done in PLINK [44]. To correct for multiple testing, 50,000 random permutations were done within each cohort, generating two empirical P-values. The first P-value was an estimate of an individual SNP’s significance, the second P-value corrected for multiple testing while preserving the correlational structure between SNPs [44]. To test for heterogeneity between the Dutch and German cohorts, a Breslow-Day test was performed in PLINK (44). Haplotype analysis was performed in Haploview v4.2 [43]. Uncorrected P-values, odds ratios (OR) and 95% confidence intervals (95% CI) are shown in Table 3. 

**Table 2:** Analysis of *MYO9B*, *PARD3* and *MAGI2* SNPs in the Dutch and German cohorts and joint analysis.

			**Initial study**			**Follow-up study**			**Joint analysis**			
			RAF patients (n = 387)	RAF controls (n > 800)	P-initial^*^	RAF patients (n = 235)	RAF controls (n = 250)	P follow-up^*^	P-joint	OR	95% CI	**P-adjusted**
rs7259292	*MYO9B*	T/C^#^	0.046	0.026	0.0053	0.047	0.020	0.0200	0.0003	1.94	1.35-2.78	**0.0031**
rs2305767	*MYO9B*	A/G	0.620	0.557	0.0021	0.590	0.607	0.5880	0.0211	0.85	0.74-0.98	0.1709^$^
rs1545620	*MYO9B*	C/A	0.448	0.364	2.3x10^-5^	0.385	0.359	0.4083	5.9x10^-5^	1.33	1.16-1.53	**0.0006**
rs1457092	*MYO9B*	A/C	0.401	0.337	0.0011	0.344	0.345	0.9807	0.0062	1.22	1.06-1.40	0.0557
rs2305764	*MYO9B*	A/G	0.433	0.381	0.0093	0.383	0.401	0.5516	0.0614	1.14	0.99-1.31	0.4193
rs10763976	*PARD3*	A/G	0.483	0.431	0.0195	0.564	0.536	0.3857	0.0157	1.19	1.03-1.38	0.1320
rs4379776	*PARD3*	A/G	0.371	0.312	0.0046	0.353	0.341	0.6995	0.0109	1.22	1.05-1.41	0.0929
rs6962966	*MAGI2*	G/A	0.493	0.458	0.1114	0.481	0.567	0.0077	0.8450	0.99	0.85-1.14	1.0^$^
rs9640699	*MAGI2*	A/C	0.390	0.384	0.7708	0.391	0.413	0.4956	0.8844	0.99	0.85-1.15	1.0
rs1496770	*MAGI2*	A/G	0.407	0.400	0.7317	0.391	0.409	0.5538	0.9575	1.00	0.86-1.15	1.0

OR, odds ratio; 95% CI, 95% confidence interval; RAF, risk allele frequency.

^#^ Risk variant/second allele

^*^ Two-tailed P-values were calculated by chi-squared test for independence of allele counts

^$^Heterogeneity between the cohorts (Breslow-Day test).

The risk variant was the associated allele in the Dutch cohort; the same variant frequencies were reported for the German cohort. A combined analysis of the Dutch and German results was performed using Cochran-Mantel-Haenszel analysis with 50,000 random permutations. This generated two P-values (P-joint and P-adjusted), an OR and 95% CI. P-joint shows an individual SNP’s significance in the combined cohort. P-adjusted was obtained after correcting for multiple testing. SNPs rs2305767 and rs6962966 showed modest evidence for heterogeneity between the Dutch and German cohort when a Breslow-Day test was performed on this data.

**Table 3:** The prevalence of *MYO9B* haplotypes in the combined Dutch and German cohorts of patients with acute pancreatitis and controls reconstructed from genotyped SNPs and their association with acute pancreatitis.

*MYO9B* haplotypes									
rs7259292	rs2305767	rs1545620	rs1457092	rs2305764	Cases (%)	Controls (%)	OR	95% CI	P-value^*^
C	G	A	C	G	460 (38)	900 (42)	1.00^‡^	–	ref
C	**A**	**C**	**A**	**A**	443 (37)	700 (33)	1.24	1.05-1.46	0.0099
C	**A**	A	C	G	190 (16)	365 (17)	1.02	0.83-1.25	0.8860
C	**A**	A	C	**A**	40 (3.3)	98 (4.6)	0.81	0.55-1.19	0.2512
**T**	**A**	**C**	C	G	52 (4.3)	50 (2.3)	2.03	1.36-3.04	0.0005

OR, odds ratio; 95% CI, 95% confidence interval.

^*^ Two-tailed P-values were calculated by chi-squared test for independence of haplotype counts.

^‡^ This haplotype was taken as the reference.

Risk alleles are in bold and underlined.

## Results

### Polymorphisms in MYO9B may increase susceptibility to acute pancreatitis

Table 2 summarizes the results for all ten SNPs across the three genes tested. A significant association was observed for the five tagging SNPs in *MYO9B* and the two variants in *PARD3* in the Dutch cohort (most significant SNP in *MYO9B*: rs1545620, p = 2.3x10^-5^; most significant SNP in *PARD3*: rs4379776, p = 0.0046; Table 2). There were nine patients with CD, IBD or type 1 diabetes mellitus in this cohort. To exclude any effect from these co-morbidities, we removed these patients from the analysis. Association analysis showed that the associations with *MYO9B* and *PARD3* remained significant in the Dutch cohort (data not shown). None of the genetic variants of *MAGI2* were associated with acute pancreatitis (Table 2). In the German cohort, an association was found for one variant in *MYO9B* and one variant in *MAGI2* (Table 2, rs7259292 and rs6962966, respectively). 

We performed a joint analysis combining the Dutch and German cohorts using the Cochran-Mantel-Haenszel method with 50,000 random permutations within both cohorts. In this analysis, four of the *MYO9B* SNPs were found to be associated with acute pancreatitis. Two of these were still associated with the disease after correcting for multiple testing (rs1545620, p = 0.0006, OR 1.33, 95%CI 1.16-1.53; rs7259292, p = 0.0031, OR 1.94, 95%CI 1.35-2.78) and one showed borderline significance (rs1457092, p = 0.0557, OR 1.22, 95%CI 1.06-1.40). Both *PARD3* SNPs were associated with acute pancreatitis, but these associations did not withstand correction for multiple testing. A Breslow-Day test showed modest evidence for heterogeneity between the two cohorts for SNPs rs2305767 in *MYO9B* and rs6962966 in *MAGI2*, but these SNPs were not significant in the final analysis (Table 2).

All SNPs in *MYO9B* were located in one haploblock and were in strong linkage disequilibrium. We therefore constructed 5-SNP haplotypes using the combined genotypes of the initial and follow-up studies (Table 3). Three of the haplotypes occurred with a frequency of more than 5% in acute pancreatitis patients or controls. The haplotype CACAA occurred more often in patients than controls (37% vs. 33%, p = 0.0099, OR 1.24, 95%CI 1.05-1.46). Of the rare haplotypes with a frequency below 5%, the haplotype TACCG occurred more often in patients than controls (4.3% vs. 2.3%, p = 0.0005, OR 2.03, 95%CI 1.36-3.04). Both of these haplotypes carry the rs1545620*C allele, which is the allele stemming from the most strongly associated SNP.

To investigate an association between the three genes tested and the course of acute pancreatitis, we did a post-hoc analysis of the prevalence of all the genetic variants in four groups of patients of the combined Dutch and German cohorts. These were patients who developed severe acute pancreatitis (n = 119), infectious complications (n = 106), infected pancreatic necrosis (n = 58), or who died (n = 20). After correcting for the number of phenotypes and SNPs, the results appeared not to be significant. We also compared patients with acute biliary pancreatitis (n = 307) to patients with acute pancreatitis with non-biliary etiology, but we found no association. 

## Discussion

We performed a candidate gene study for *MYO9B*, *PARD3* and *MAGI2* looking for susceptibility to acute pancreatitis. All three genes are thought to influence intestinal permeability [21,23,25]. By analyzing a combined cohort of Dutch and German patients with acute pancreatitis, we found an association of two genetic variants in *MYO9B* for susceptibility to this disease. The SNP with the strongest association was rs1545620 (p = 0.0006, OR 1.33, 95%CI 1.16-1.53), which is a non-synonymous variant leading to an amino acid change [25]. This SNP was very strongly associated (p = 2.3x10^-5^, Table 2) in the Dutch cohort, but not in the German cohort. The differential association could not be attributed to heterogeneity between the cohorts. 

Our analyses in two separate cohorts resulted in different findings. In the Dutch cohort, all five variants in *MYO9B* were associated with acute pancreatitis, but we were surprised to see that only one of these SNPs showed association in the German cohort. The *MAGI2* SNP rs6962966 did show heterogeneity between cohorts and did show a different pattern of association between the Dutch and German cohort, with the latter providing modest evidence for association (uncorrected p = 0.0077). While statistical power is one explanation for these differences, our findings highlight the need to replicate such results before accepting them.

Intestinal permeability is a critical factor for the course of acute pancreatitis, since a breakdown of the barrier function enables bacterial translocation, which may subsequently cause infectious complications [13-16]. We therefore explored whether the genetic variants had any relationship with the severity of disease (severe vs. mild acute pancreatitis), mortality, or the occurrence of infectious complications. These analyses revealed no associations. 

One of the strengths of our study is the size of the combined cohort: 622 patients for whom clinical data were available. Most previous genetic association studies in acute pancreatitis consisted of quite small patient populations (n = 35-470). Yet, despite our relatively large cohort, our subgroup analyses did not reveal any convincing results. Future studies will need to investigate the genotypes in subgroups of patients, e.g. in those with severe acute pancreatitis. The clinical classification, however, of patients with severe acute pancreatitis into subgroups is subjective and heterogeneous, which could also account for the lack of association between genetic variants and clinical course. Finally, there could be other genetic or environmental factors that determine the course of acute pancreatitis.

The *MYO9B* gene has consistently been found to be associated with IBD in cohorts from different countries [21,23,25-28]. The rs1545620 SNP with the highest OR is a non-synonymous SNP inducing an amino acid change (Ala1011Ser) in the neck region of the MYO9B protein; it is necessary for the motor activity of MYO9B on actin filaments [30,31]. A conformational change of the protein could therefore result in lower MYO9B activity. This could lead to a diminished capacity for maintaining tight junction and cytoskeleton structure.

The association of variants of *MYO9B* with acute pancreatitis points to a possible shared genetic mechanism that impairs mucosal barrier function not only in acute pancreatitis, but also in CD, IBD and type 1 diabetes mellitus. We found polymorphisms of a gene likely to be involved in maintaining tight junction function (and potentially gastrointestinal permeability) to be associated with susceptibility to acute pancreatitis rather than to the clinical course of the disease. This runs contrary to current knowledge on the pathophysiology of acute pancreatitis and we have no biological explanation for our observation. Unfortunately, there are no functional data on the role of gastrointestinal permeability and the development of acute pancreatitis. Our findings should therefore lead to experimental studies to elucidate this new, potentially important, pathophysiological concept in acute pancreatitis. 

We have shown that *MYO9B* may be involved in acute pancreatitis, possibly due to its potential role in regulating the intestinal barrier function. Our results open the way to thinking about shared mechanisms leading to mucosal barrier impairment. The presence of genetic variants of *MYO9B* in an individual may be the first step that can lead to different diseases, depending on subsequent events. Whether these different outcomes are influenced by environmental factors (such as in acute pancreatitis) or by other sets of modifier genes (such as in celiac disease and inflammatory bowel disease) still needs to be determined. 
